# The Role of Copy Number Variation in Susceptibility to Amyotrophic Lateral Sclerosis: Genome-Wide Association Study and Comparison with Published Loci

**DOI:** 10.1371/journal.pone.0008175

**Published:** 2009-12-04

**Authors:** Louise V. Wain, Inti Pedroso, John E. Landers, Gerome Breen, Christopher E. Shaw, P. Nigel Leigh, Robert H. Brown, Martin D. Tobin, Ammar Al-Chalabi

**Affiliations:** 1 Departments of Health Sciences and Genetics, University of Leicester, Leicester, United Kingdom; 2 MRC Centre for Neurodegeneration Research, Institute of Psychiatry, King's College London, London, United Kingdom; 3 Day Neuromuscular Research Laboratory, Department of Neurology, University of Massachusetts Medical School, Worcester, Massachusetts, United States of America; 4 MRC Centre for Social, Genetic and Developmental Psychiatry, Institute of Psychiatry, King's College London, London, United Kingdom; 5 NIHR Biomedical Research Centre for Mental Health, South London and Maudsley NHS Foundation Trust and Institute of Psychiatry, King's College London, London, United Kingdom; University of Giessen, Germany

## Abstract

**Background:**

The genetic contribution to sporadic amyotrophic lateral sclerosis (ALS) has not been fully elucidated. There are increasing efforts to characterise the role of copy number variants (CNVs) in human diseases; two previous studies concluded that CNVs may influence risk of sporadic ALS, with multiple rare CNVs more important than common CNVs. A little-explored issue surrounding genome-wide CNV association studies is that of post-calling filtering and merging of raw CNV calls. We undertook simulations to define filter thresholds and considered optimal ways of merging overlapping CNV calls for association testing, taking into consideration possibly overlapping or nested, but distinct, CNVs and boundary estimation uncertainty.

**Methodology and Principal Findings:**

In this study we screened Illumina 300K SNP genotyping data from 730 ALS cases and 789 controls for copy number variation. Following quality control filters using thresholds defined by simulation, a total of 11321 CNV calls were made across 575 cases and 621 controls. Using region-based and gene-based association analyses, we identified several loci showing nominally significant association. However, the choice of criteria for combining calls for association testing has an impact on the ranking of the results by their significance. Several loci which were previously reported as being associated with ALS were identified here. However, of another 15 genes previously reported as exhibiting ALS-specific copy number variation, only four exhibited copy number variation in this study. Potentially interesting novel loci, including *EEF1D*, a translation elongation factor involved in the delivery of aminoacyl tRNAs to the ribosome (a process which has previously been implicated in genetic studies of spinal muscular atrophy) were identified but must be treated with caution due to concerns surrounding genomic location and platform suitability.

**Conclusions and Significance:**

Interpretation of CNV association findings must take into account the effects of filtering and combining CNV calls when based on early genome-wide genotyping platforms and modest study sizes.

## Introduction

Amyotrophic lateral sclerosis (ALS) is a neurodegenerative disease of motor neurons resulting in progressive weakness and death, usually within about 3 to 5 years of diagnosis as a result of neuromuscular respiratory failure. The lifetime risk of ALS is about 1 in 400[1] with peak incidence in the sixth decade but the causes remain largely unknown. In about 10% of cases there is a family history. Following linkage, mutations causing familial ALS have been identified in the genes *SOD1* [2]–[4], *TARDBP* [5], [6], and *FUS(TLS)* [7] accounting for about 20 to 30% of cases, and *ALS2* [8]–[10], *SETX* [*11*]–[13**], and *VAPB* [14] accounting for some atypical, rare forms.

Attempts to identify genetic causes for the common sporadic form of ALS have met with limited success. Candidate gene analyses have identified *NEFH* deletions and insertions[15]–[17], and *ANG* mutations[18]–[22] as possible risk factors, and both have also been identified in affected individuals from families with ALS. Genome-wide association studies have not found a single consistent risk variant but *ITPR2* [23], *DPP6* [24] and UNC13A[25] variants have been identified in some studies, and *KIFAP3* identified as a survival gene[26]. The numbers studied are still in the low thousands and research is therefore continuing.

Copy number variation of the *SMN* gene, responsible for spinal muscular atrophy, has been implicated as a risk factor for sporadic ALS[27]. Two previous studies have examined copy number information derived from genome-wide association studies of ALS[28], [29] concluding that multiple rare copy number variants (CNVs) may be a more important risk factor for sporadic ALS than common CNVs. A study utilising data from a combined total of 651 ALS cases and 625 controls, genotyped on either the Illumina 300K or the Illumina 550K SNP chip, identified 39 loci that were highlighted as containing rare ALS-specific copy number changes[28]: plausible candidate regions within this category included an ataxin gene locus and the hereditary haemochromatosis locus. In the same study and a previous study based on a subset of the same data (406 ALS cases and 404 controls)[29], nominally significant associations between CNVs that were not ALS-specific and risk of ALS were found for a total of 25 loci. However, none of these associations remained after Bonferroni correction for multiple testing.

Sophisticated algorithms are available to mine SNP genotyping data for CNVs[30]–[32]. However, many SNPs in CNV regions have been excluded from some genome-wide genotyping platforms due to the effect of copy number variation on Hardy-Weinberg equilibrium and Mendelian inheritance[33]. Boundary uncertainty due to low resolution of the platform can lead to CNV calls that arise from underlying neighbouring or nested, but distinct, CNV events being clustered together across individuals as representing the same CNV[33]. The challenge lies in accurately combining calls that may not be identical across individuals but which arise as the result of the same underlying signal and distinguishing calls that overlap because of boundary uncertainty. In addition, it is known from the study of genomic disorders that overlapping or nested CNVs can give rise to distinct phenotypic characteristics [34]–[36]. Therefore, an approach which combines any overlapping CNV calls could lead to misclassification of CNV regions. In addition to sample size and genome coverage, the misclassification of CNV regions could explain the thus far weak associations with CNVs found for ALS.

In this study, CNV calls derived from Illumina HumanHap 300 v.1.0 SNP array intensity data from an initial sample size of 730 ALS cases and 789 controls were analysed for association with ALS. CNV calls were merged into distinct, nested and overlapping CNV regions for association analysis. A gene-based association approach was also used for comparison and to test the hypothesis that multiple, rare CNVs affecting a subset of genes and pathways may contribute to ALS risk. We compared our findings with those of previous studies.

## Results

### Genome-Wide CNV Detection: Raw CNV Calls

Following the sample quality control filters, 621 cases and 675 controls remained for further analysis (age and sex details of the cases and controls are given in Table 1). A total of 80023 unfiltered CNV calls were made from the data. These calls were filtered according to log Bayes factor (posterior measure of confidence in the call) and proximity to the centromere and CNV calls from individual samples with an extreme number of CNV calls across their genome were also excluded (see Methods). To facilitate comparison with published findings we did not exclude calls within the telomeric band (but loci containing these calls are flagged where appropriate in subsequent sections of the results). Following these filters, a total of 11321 CNV calls across 1194 samples (14 of which contained no CNVs after filtering) remained. The total number, mean numbers per individual and median sizes of each type of CNV are given in Table 2. A two-tailed Mann Whitney U-test was used to compare the mean number of CNVs per individual and the median size of the CNVs of each type between cases and controls. ALS cases were found to contain significantly more duplications (p = 0.0018) and significantly larger duplications (p<0.001) than controls.

**Table 1:** Sex and age of sample population following sample quality control.

	Case			Control			Overall		
	n	Mean age	Age range	n	Mean age	Age range	n	Mean age	Age range
**Male**	**392**	52.0	21–86.5	**389**	62.4	25–95	**781**	57.2	21–95
**Female**	**229**	56.9	19–83	**286**	54.9	21–89	**515**	55.8	19–89
**Total**	**621**	53.8	19–86.5	**675**	59.2	21–95	**1296**	56.6	19–95

**Table 2:** Overall summary of CNV calls across 1194 (575 cases and 619 controls) individuals.

Type		Cases	Controls	P value
Duplications	Total	4015	3941	
	Mean per individual (range)	7.03 (0–21)	6.44 (0–21)	**0.0018**
	Median size, kb (range)	202.3 (0–3074)	160.3 (0–10000)	**<0.0001**
Heterozygous deletions	Total	1416	1685	
	Mean per individual (range)	1.13 (0–6)	1.12 (0–2)	0.4115
	Median size, kb (range)	47.6(0.57–1513)	46.8 (0.35–9888)	0.1127
Homozygous deletions	Total	128	136	
	Mean per individual (range)	2.77 (0–14)	2.99 (0–16)	0.2247
	Median size, kb (range)	5.38(0–425)	5.5(0–383)	0.8699
Total		5559	5762	

Significant p values are given in bold.

### Genome-Wide CNV Association: By CNV Region

CNV calls that passed the filters described above from across all individuals were merged into CNV loci for association testing (see Methods). This region-based approach enables consideration of boundary estimation uncertainty and the possibility of distinct but nested or overlapping CNV loci. A reciprocal overlap threshold of >70% was used initially and a total of 2983 CNV loci (median size 72.9 kb) across the genome were identified. Of these, 119 (median size 90.6 kb) were observed to be ALS-specific (i.e. present in 2 or more ALS cases but not observed in any controls) and 136 (median size 79.1 kb) were observed to be control-specific (i.e. in 2 or more control samples but not observed in any cases) (Table 3). There was no significant difference between the number of ALS-specific CNV loci and the number of control-specific CNV loci either overall or by CNV type (heterozygous deletions, homozygous deletions, duplications, multiallelic [loci consisting of both duplications and deletions]). Five loci were found to contain both heterozygous and homozygous deletion calls – all five of these CNV regions were identified only in controls and none were found in cases (p = 0.03). There was no significant difference between the size distribution of ALS-specific or control specific heterozygous deletions, homozygous deletions or duplications. The control-specific multiallelic loci were found to be significantly larger (p = 0.028) than the ALS-specific multiallelic loci (median sizes of 395.1 kb and 49.5 kb, respectively). One hundred and thirty CNV loci were observed in more than 1% of samples and 24 were identified in more than 5% of samples.

**Table 3:** ALS-specific and control-specific CNV counts.

Type	ALS-specific (n)	Control-specific (n)	P value*	ALS-specific: median size (kb)	Control-specific: median size (kb)	P value†
All	119	136	0.69	90.6	79.1	0.52
Heterozygous deletions	50	50	0.68	53.9	49.8	0.48
Homozygous deletions	5	4	0.65	2.4	23.4	0.62
Duplications	59	68	0.74	158.7	103.0	0.14
Multiallelic	5	9	0.36	49.5	395.1	**0.028**
Deletion (het or hom)	0	5	**0.032**	na	8.3	na

ALS-specific CNVs defined as those present in 2 or more ALS cases but in no controls. Control-specific defined as those present in 2 or more controls but no ALS cases.

* Pearson's chi-squared test, 1 df.

† Mann-Whitney U-test.

Of the 2839 autosomal loci (defined from CNV calls using a reciprocal overlap threshold of >70%) tested for association with ALS, 31 showing gains in copy number were associated with ALS (uncorrected nominal p<0.05, Table 4 and Table S1 and Table S2). Loci within the telomeric band are flagged and the proximity of each loci to the centromeres is indicated in Table 4. Only the top locus on chromosome 5 was associated after Bonferroni correction for multiple testing. Of these, 26 were found at a frequency of greater than 1% in the study population with 9 of these having a frequency of greater than 5% (although all but one, the *RDH13* region, map very close to the centromere: a region believed to be prone to false positive CNV calls). Eleven of the thirty-three “gains” loci overlapped at least one Copy Number Polymorphism (CNP), identified by McCarroll and colleagues[37] as copy number loci with a frequency of >1% in the HapMap population. Four CNV loci showing loss of copy number were associated with ALS (nominal p<0.05). Only one of these “loss” loci was present at a frequency of greater than 1% and this locus also overlapped a CNP. Finally, two further loci showing both losses and gains in copy number across the study population were associated with ALS. One of these two loci was present at a frequency of greater than 1% in the study population and also overlapped a CNP.

**Table 4:** Summary of association results with p<0.01 (using Fishers exact test).

Chr	Start (bp)	End (bp)	ALS	controls	P value	Distance from centromere (kb)	Overlapped genes	CNP^1^	Frequency across all samples
***Gains***									
5	45850032	46384240	264	174	1.07×10^−9^	324.3	-	2	0.367
8	47062007	47406312	30	8	8.21×10^−5^	276.1	-	0	0.032
12	36528296	36801139	157	109	2.65×10^−4^	521.8	-	1	0.223
19	32615675	32935836	165	122	5.87×10^4^	2852.1	RDH13	0	0.240
7	61663407	62155064	172	132	0.0018	851.0	-	0	0.255
3	33270957	33296620	3	18	0.0027	57303.8	*FBXL2*	0	0.018
8	47062007	47711911	3	18	0.0032	428.9	-	1	0.018
8	43689385	43910848	74	52	0.0046	157.9	-	0	0.106
16	969913	1834962	8	15	0.0056	33740.9#	SOX8, SSTR5, C1QTNF8, **CACNA1H**, TPSG1, TPSB2, TPSAB1, TPSD1, **UBE2I**, **BAIAP3**, C16orf42, GNPTG, UNKL, C16orf91, CLCN7, C16orf38, TELO2, IFT140, TMEM204, CRAMP1L, HN1L, **MAPK8IP3**, NME3, MRPS34, EME2, **SPSB3**, NUBP2, **IGFALS**, HAGH, FAHD1, C16orf73	1	0.019
5	28842013	28912873	7	0	0.0058	17563.9	-	0	0.006
4	761587	1014752	16	4	0.0068	48466.7#	**CPLX1**, GAK, TMEM175, DGKQ, *IDUA*, *SLC26A1, FGFRL1*	0	0.017
***Losses***									
22	21011312	21394287	0	11	0.002	6872.8	*ZNF280B, ZNF280A, PRAME, BCR,* GGTLC2,	0	0.009

A reciprocal overlap threshold of >70% was used.

1 Number of CNPs from the McCarroll CNP map[37] that also overlap this region.

# Regions that were within the telomeric chromosome band. Seven additional regions identified to show significant association with ALS were unable to be mapped to build 36 of the human genome. These regions are given in table S1. Genes which were also identified with p<0.01 in the gene-based analysis are given in italics. Genes which may be reasonable ALS candidates are in bold.

### Sensitivity Analyses

For each of the loci defined using a 70% reciprocal overlap that reached p<0.05, the raw CNV calls within these regions were re-assigned to CNV loci using different reciprocal overlaps (>0%, >50% or 100%). Where re-assignment resulted in more than one CNV locus within the region defined using 70% overlap, the locus giving the highest significance was included(Table 5). The choice of reciprocal overlap threshold used has an effect on the order of significance of the loci with some loci reaching smaller p values if different thresholds are used while others give a higher p value or are lost as nominally significant loci. Although the “top” locus on chromosome 5 retained a very low p value for all thresholds, the second and third ranking loci gave p values of >0.01 if any overlap (>0%) or a >50% threshold was used. Three loci that achieved a p value<0.05 when a 70% reciprocal overlap was used were lost if any of the other overlap thresholds were used to combine the raw calls.

**Table 5:** Sensitivity analyses.

chr	start	end	P value (70%)	>0%	>50%	100%
Gains						
5	45850032	46384240	1.07E-09	**	**	**
8	47062007	47406312	8.21E-05	-	*	**
12	36528296	36801139	0.000265	*	*	**
19	32615675	32935836	0.000587	*	**	**
7	61663407	62155064	0.0018	**	**	**
3	33270957	33296620	0.0027	**	**	*
8	47062007	47711911	0.0032	-	*	**
8	43689385	43910848	0.0046	-	*	**
16	969913	1834962	0.0056	-	*	*
5	28842013	28912873	0.0058	*	**	-
4	761587	1014752	0.0068	**	**	*
5	510955	738748	0.012	-	-	-
20	60320976	60493125	0.016	**	**	-
19	32615675	32851754	0.022	*	**	**
6	58675121	58878583	0.024	**	**	**
19	60270514	60293927	0.025	*	**	*
14	103232016	103721150	0.025	-	*	-
20	60214968	60493125	0.035	**	**	-
16	1744358	1781034	0.038	-	*	*
14	104197399	104356204	0.043	-	-	-
8	142512205	142529990	0.046	-	-	-
3	101837214	101916282	0.047	*	*	*
21	43646295	43663581	0.047	*	*	**
14	19375271	19536664	0.049	*	*	-
11	382079	434628	0.049	**	*	*
Losses						
22	21011312	21394287	0.002	*	**	-
10	82869699	82882268	0.013	**	*	*
11	539119	652407	0.025	**	*	-
17	43969101	44059535	0.032	-	*	-
Losses/gains						
8	47224322	47711911	0.003	-	*	**
8	144686338	144765210	0.012	**	**	-
						

For each of the loci defined using a 70% reciprocal overlap that reached p<0.05, the raw CNV calls within the regions were re-assigned to CNV loci using different reciprocal overlaps (>0%, >50% or 100%). Where re-assignment resulted in more than one CNV locus within the region defined using 70% overlap, the locus giving the highest significance was included.

** p value<0.01,

* p value<0.05, - p value>0.05.

### Genome-Wide CNV Association: By Gene

In addition to the region-based approach described above, a gene-based association analysis was also undertaken to accommodate the hypothesis that multiple rare CNVs may contribute to ALS risk by affecting one or a subset of key genes. The numbers of CNV calls that overlap each gene (at least 1 base pair overlap) were counted and the difference between the number called in cases and the number called in controls per gene was tested.

**Figure 1:**
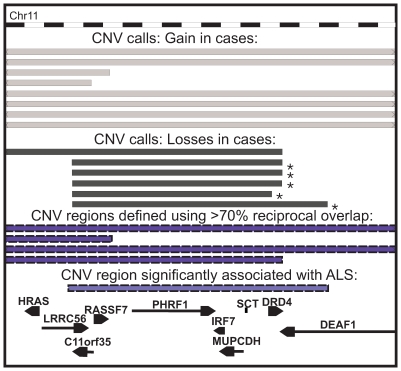
All CNV calls and CNV regions overlapping CNV region chr11: 539119-652407. Raw CNV calls are shown in dark grey. No control losses or gains were detected within the region shown but 8 gains in cases and 6 losses in cases were detected. Blue bars represent CNV regions defined by merging CNV calls that share a greater than 70% reciprocal overlap. The purple bar represents the CNV region found to show significant association in this study: the CNV calls that were merged into this CNV region are indicated with an asterisk. Genes are shown at the bottom. Genomic position is shown along the top.

Fifty of the 102 genes identified as being associated (p<0.05) with ALS using the gene-based approach were also identified using the region-based approach (Table 6). Twenty-eight of these genes were significantly associated with ALS using a gene-based approach at a significance level of p<0.01. The 8 most significant genes were all located within the chromosome 11 region (build 36 coordinates – chr11:539119-652407) which showed ALS-specific loss in copy number in 5 samples (this region was also found to show significant association with ALS if different reciprocal overlap thresholds were used to merge the data into CNV regions, but with gains in copy number showing association). Figure 1 shows the raw CNV calls and how these are clustered into CNV regions at this locus.

**Table 6:** Results of gene-based association approach.

Gene	Pooled gains and losses			Losses			Gains		
	Cases	Controls	P value	Cases	Controls	P value	Cases	Controls	P value
C11orf35	14	0	3.96E-05	8	0	0.002988	6	0	0.01271
LRRC56	14	0	3.96E-05	8	0	0.002988	6	0	0.01271
RASSF7	13	0	8.13E-05	7	0	0.006159	6	0	0.01271
MUPCDH	12	0	0.000167	6	0	0.01271	6	0	0.01271
PHRF1	12	0	0.000167	6	0	0.01271	6	0	0.01271
IRF7	12	0	0.000167	6	0	0.01271	6	0	0.01271
SCT	12	0	0.000167	6	0	0.01271	6	0	0.01271
DRD4	11	0	0.000343	6	0	0.01271	5	0	0.026252
**EEF1D**	10	0	0.000705	7	0	0.006159	3	0	0.112288
NAPRT1	9	0	0.00145	6	0	0.01271	3	0	0.112288
C8orf73	9	0	0.00145	6	0	0.01271	3	0	0.112288
GSDMD	9	0	0.00145	6	0	0.01271	3	0	0.112288
FBXL2	4	21	0.001771	4	21	0.001771	0	0	na
BCR	8	26	0.004875	1	4	0.375881	7	22	0.013279
C14orf2	7	0	0.006159	7	0	0.006159	0	0	na
**PPP1R13B**	7	0	0.006159	7	0	0.006159	0	0	na
DEAF1	7	0	0.006159	6	0	0.01271	1	0	0.482008
LAMA5	27	56	0.006206	27	56	0.006206	0	0	na
PRAME	9	27	0.006383	2	5	0.454278	7	22	0.013279
FGFRL1	19	6	0.007514	19	6	0.007514	0	0	na
IDUA	19	6	0.007514	19	6	0.007514	0	0	na
SLC26A1	19	6	0.007514	19	6	0.007514	0	0	na
GATA5	3	15	0.008199	3	15	0.008199	0	0	na
ZNF280B	9	26	0.009548	2	4	0.6881	7	22	0.013279
ZNF280A	9	26	0.009548	2	4	0.6881	7	22	0.013279

Results are ranked by p-value when gains and losses are pooled together. A 2-sided Fishers exact test was used to test for association. Genes which we considered reasonable ALS candidates are in bold.

### Gene Set and GRAIL Analysis

Gene Set Analysis (GSA) and GRAIL analyses were carried out on the CNV regions and ALS-specific/control-specific CNV calls, respectively, to identify any gene-sets that might contain ALS-associated copy number variation. For GSA, no gene-set was associated with ALS using a significance threshold of p<5.74×10^−4^ (Bonferroni corrected). Table S3 shows all pathways with p<0.05. Four of these nominally significant pathways are involved in apoptosis and cell death, one in muscle organ development (identified using both region-based and gene-based p-values) and one in central nervous system development. No significantly associated genes were identified from the ALS-specific CNV calls using GRAIL but five genes significantly associated with the control-specific CNV calls were identified (Table S4).

### Candidate Regions/Previously Detected Regions

Regions that were previously identified to show association with ALS were investigated in our data. ALS-specific gains and losses were observed in 6 ALS cases at the *GSDMD* gene region (association p value = 0.012). This region was also previously identified by Cronin and colleagues[28] as containing ALS-specific gains and losses. *GSDMD* was also found to be significantly associated (p<0.01) with ALS in our gene-based approach. Of the other 15 genes identified to exhibit ALS –specific copy number variation by Cronin and colleagues[28] only four were also found to exhibit copy number variation in this study but in both cases and controls and with no significant differences between them using either approach.

A region 473.4 kb downstream of *SH2D4B* region on chromosome 10 showed association with ALS by loss of copy number in this study, using a region-based approach, with 7 ALS cases and 22 controls containing a loss at this locus. A significant association with ALS with losses in 9 ALS cases and 21 controls at the *SH2D4B* locus was reported by Cronin and colleagues[28].

Three further loci that were identified as showing significant association with ALS in this study were also identified as significantly associated with ALS by Cronin and colleagues[28]: the chromosome 5 locus flanking *HCN1*, the chromosome 8 locus flanking *POTE8* and the chromosome 11 locus including *OR4A5* and flanking *OR4C12* (this locus was lost from our region-based approach upon conversion of genome coordinates to build 36, see table S1). However, the direction of effect was different in this study with significant differences found between the numbers of ALS cases and controls with gains of copy number at these loci (losses of copy number at these loci were reported in the previous study).

## Discussion

With a sample size of 1194 (575 ALS cases and 621 controls) and genotypes across more than 317,000 SNPs, we set out to investigate the role of copy number variation in ALS using a widely available CNV calling algorithm. We used two different approaches to collate and test association of the raw CNV calls with ALS and compared our results with those of previous studies.

Although none of the loci identified as being associated with ALS contained, or were close to, any genes previously reported as plausible ALS candidate genes [38], [39], several potential new genes of interest were highlighted. The region on chromosome 16 encompasses several genes that are reasonable candidates for ALS susceptibility. These have functions in calcium signalling (*CACNA1H*), axonal transport, (*MAPK8IP3*), nerve growth factors (*IGFALS*), angiogenesis (*BAIAP3*), and the ubiquitin proteasome system (*UBE2I, SPSB3*). The *CPLX1* gene on chromosome 4 encodes a protein involved in synaptic vesicle exocytosis. The *PPP1R13B* gene on chromosome 14 is an apoptosis stimulating protein. Perhaps the most interesting potential ALS candidate of the genes identified here is *EEF1D*, a eukaryotic translation elongation factor 1 delta on chromosome 8, which encodes a subunit of the elongation factor-1 complex, responsible for the enzymatic delivery of aminoacyl tRNAs to the ribosome. This process has been directly implicated in genetic studies of motor neuron diseases (*GARS* [40], [41], *YARS* [42] and *IARS* [43] genes), and the RNA processing pathway in general is involved in amyotrophic lateral sclerosis as evidenced by ALS-linked mutations identified in the *SETX, FUS* and *TARDBP* genes[5]–[7], [11]–[13], and the association of ALS with variants in the *ANG* [18]–[22] and *ELP3* [44] genes.

Although we found few differences between the numbers and sizes of ALS-specific and control-specific regions overall, we identified 5 regions that were ALS-specific and achieved significance (before multiple testing correction). Many of the genes in these regions were also associated when the raw CNV calls were tested using a gene-based approach, including *EEF1D* and *PPP1R13B*. Although we found the copy number variation at a chromosome 11 region to be ALS-specific in our sample population, CNVs at this locus in healthy individuals have been recorded previously [45] and are recorded in the Database of Genomic Variants[46] suggesting that this region may not be ALS-specific.

There was a small amount of overlap between the results from this study and those of a previous study investigating the role of CNV in ALS risk but the majority of the findings of the previous study were not replicated in our data. Cronin et al[28] found a significant difference between the median size of heterozygous deletions in cases and controls. In this study, the median size of heterozygous deletions was larger in cases compared to controls but this difference was not found to be significant. When our data was filtered using an alternative set of filters that were closer to those implemented by Cronin et al[28] (see Appendix S1), the size of heterozygous deletions were found to be larger in cases than in controls but the difference did not reach significance (p<0.05). Significant (P<0.01) differences between the mean number per individual and the median size of duplications were found between cases and controls using both our original filters and those similar to Cronin et al[28].

Our primary analysis was undertaken using a region-based approach which aimed to reduce the impact of errors in boundary estimation. Four different reciprocal overlap thresholds were applied to the data to merge CNV calls into CNV regions and, of the 38 loci showing association with ALS if a >70% reciprocal overlap was used, all but 3 regions were also associated with ALS (nominal P<0.05) if a different reciprocal overlap threshold was used (i.e. any overlap, >50% overlap and 100% overlap). However, the level of significance achieved varied for most regions according to the overlap threshold used; if validation were to be undertaken for a limited number of regions, the choice of such regions would be dependent on the choice of reciprocal overlap threshold used. Interestingly, many of the regions identified using the region-based approach, were also represented amongst the hits from the gene-based analysis. The potentially interesting genes within the chromosome 16 region and CPLX1 were not significantly associated with ALS under the gene-based approach. However, *EEF1D* and *PPP1R13B* were significantly associated with ALS using both approaches.

No evidence of any gene set being overrepresented amongst those affected by ALS-specific copy number variation was found by GRAIL analysis. There were five significant genes amongst those affected by control-specific copy number variation, identified by GRAIL analysis, including GLL1 which is involved in B cell biology and has been associated with agammaglobulinemia[47]. No significantly associated gene-sets were identified using GSA although several involving cell death, muscle organ development and central nervous system development were amongst those showing nominal significance (uncorrected p<0.05). If a result that is significant in a CNV genome-wide association study is not significant in GSA it might still be important if it perturbs a pathway. For example, a deletion upstream of the *IRGM* gene affects the expression of IRGM but the gene itself is not believed to be copy number variable [48]. In this case GSA using CNV association data would not report the IRGM-related pathways.

Previous studies have found limited evidence for a role of CNV in sporadic ALS hypothesising that multiple rare variants are more likely to contribute to risk than common variants. In this study, although nine copy number variable regions found in more than 5% of the study population showed association with ALS, seven of these were found to map close to the centromeres and may be artefactual. In addition, the regions on chromosomes 4, 8, 14 and 16 which are reported here as being associated with ALS and containing potential ALS candidates all lie within the telomeric chromosome band and so must also be treated with caution. The telomeric and centromeric regions may be more prone to false CNV calls than other regions of the genome, in part due to the lower density coverage of older genotyping arrays in these regions. Findings based on newer generation SNP arrays (which also include non-SNP copy number variation probes) have shown strong evidence of common CNVs in these regions [37] suggesting that there may be true copy number variants in these regions although caution must be taken when studying these regions on older platforms. The gene-based approach also reported genes from within some of the region-based approach findings, including the regions on chromosomes 8 and 14 which contain potential ALS candidate genes. The chromosome 11 region at which ALS-specific copy number variation was observed was reported using both approaches (with p<0.01 for each gene using gene based approach and p = 0.025 using region-based approach, although this region also lies within 1.5 Mb of the telomere and therefore must be treated with caution). Evidence of association in regions for which copy number variation has previously been characterised by McCarroll and colleagues [37] suggests that a role of common copy number variation in ALS risk should not be ruled out. It should be noted that the HumanHap300 chip has low (or no) coverage of regions known to contain common copy number variants and further studies involving larger sample sizes and using denser SNP arrays or SNP-CNV hybrid arrays should be undertaken to further investigate the role of CNV in ALS and provide replication of the findings presented here.

## Methods

### Ethics Statement

This study was conducted according to the principles expressed in the Declaration of Helsinki. The study was approved by the Institutional Review Board of Massachusetts General Hospital. All patients provided written informed consent for the collection of samples and subsequent analysis.

### Participants

Study participants were individuals of European ancestry attending the ALS Clinic of the Massachusetts General Hospital, Boston, MA, USA. All cases fulfilled the El Escorial criteria for Definite or Probable ALS. Controls were spouses or others attending with the patient (all unrelated), or blood donors from the same geographical region and matched for age. Patients with a family history of ALS were excluded.

DNA was extracted from whole blood using standard methods after written informed consent. Genotyping was performed with Illumina BeadArrays at the Broad Institute in Boston (HumanHap300 v.1.0).

### CNV Calling

**Figure 2:**
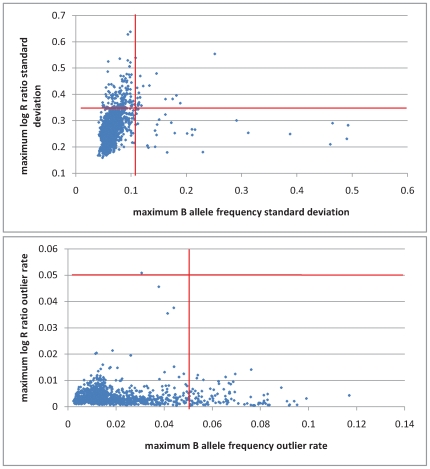
Sample quality measures (log R ratio and B allele frequency outlier rates and standard deviations). Maximum LRR standard deviation against maximum BAF standard deviation (top) and maximum LRR outlier rate against maximum BAF outlier rate (bottom). Thresholds for exclusion are shown in red.

Log R ratios and B allele frequencies for 1519 samples, including 730 ALS cases and 789 control samples, across all 317503 genotyped SNPs were calculated using BeadStudio v3.0 software (Illumina, San Diego, CA, USA). QuantiSNP [32] was used to screen the genotyping data for CNVs. One sample failed to run due to at least 26,000 missing SNPs and was excluded. All remaining samples were filtered for quality using thresholds of the log R Ratio (LRR) and B allele frequency (BAF) outlier rates and standard deviations recommended by the program authors[32]. Samples with LRR or BAF outlier rates of >0.05 or with log R ratio or B allele frequency standard deviations of >0.35 or >0.1, respectively, following GC correction were excluded (Figure 2). Following these sample quality control filters, 621 cases and 675 controls remained. A total of 80023 unfiltered CNV calls were made from the data.

### Filtering CNV Calls

In order to minimise the number of false positive CNV calls without compromising the sensitivity of detection of true CNVs, we undertook simulations to define appropriate filtering thresholds (see Appendix S1 and Figure S1). QuantiSNP provides a log Bayes factor (LBF) value for each CNV call, which is a posterior measure of confidence in the call. From the simulations, a log Bayes factor threshold of 6 was chosen (figure S1). A total of 60596 calls were found to have a log Bayes factor of less than 6 and were excluded. Although it was found that there was very low sensitivity for detecting small CNVs, no length filters were chosen as filtering on length was found to decrease the sensitivity of the method to detect longer CNVs.

Any calls that overlapped the centromeres (coordinates obtained from the relevant version of the UCSC genome browser database) were excluded (625 out of 19427). As a major aim of our study was to enable independent comparison with previously published loci, we did not exclude CNV calls which were within the telomeric chromosome band. Samples that were outliers in terms of the number of CNV calls across their genome were excluded along with their calls (7481 out of 18802 calls and 102 samples). The threshold for this was calculated using the upper quartile+1.5x(IQR) [28], [29].

### Defining CNV Regions

**Figure 3:**
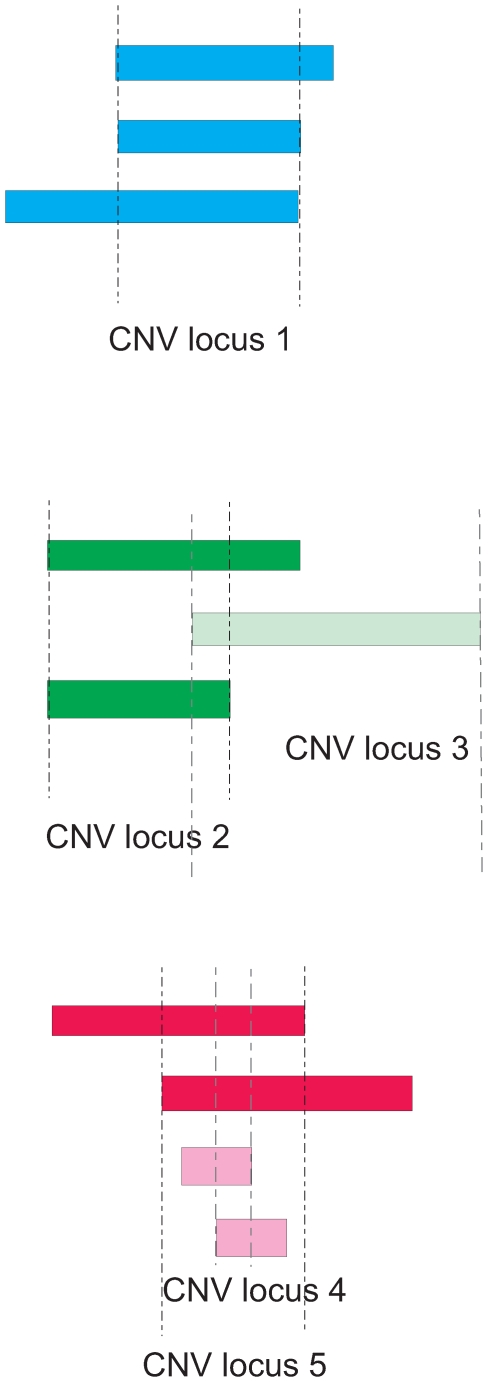
Clustering CNV calls into CNV loci based on a reciprocal overlap threshold of 50%. Each coloured bar represents one CNV call in a single individual (note: the method used here cannot distinguish overlapping calls in the same individual). CNV loci are defined by vertical dashed lines. CNV locus 1 shows three CNV calls that each share a greater than 50% reciprocal overlap with each of the other CNV calls at that locus. Overlapping CNV loci 2 and 3 result from three overlapping CNV calls, of which only two share a reciprocal overlap of greater than 50%. CNV locus 4 and CNV locus 5 are an example of how nested CNVs can occur.

CNV calls were clustered into CNV loci using a reciprocal overlap threshold (Figure 3). A threshold of >70% (pairs of CNV calls being compared must each be over at least 70% of the other CNV) was chosen. Thresholds of >0% (i.e. any overlap), >50% and 100% were also tested (sensitivity anal ysis) to investigate the effect this has on the association testing results under the rationale that combining CNV calls must also take into account the biological phenomenon of nested or overlapping CNVs along with the uncertainty of boundary estimation by the method used.

CNV calls were first sorted by length (number of SNPs). The largest CNV call was assigned the initial CNV locus identifier. Each subsequent CNV call was then compared to all CNV calls previously assigned to a CNV locus and was in turn allocated to the CNV locus for which it showed a reciprocal overlap of greater than the chosen threshold with all CNV calls already allocated to that locus. If it did not meet the overlap criteria for all CNV calls currently assigned to any of the previously designated CNV loci, it was assigned to a new CNV locus. This allows nested CNV loci to be designated and prevents CNV loci from “spreading” such that they contain CNV calls that do not overlap one another.

### Defining Gene Overlap for Gene-Based Association

The coordinates of all 11321 CNV calls that passed the filters above were mapped from Build 35 to Build 36 of the human genome (Batch Coordinate Conversion tool, UCSC Genome Bioinformatics Group) in order to extract the number of calls that overlapped each gene (Table Browser tool, UCSC Genome Bioinformatics group). A total of 101 unique CNV call locations, comprising 1197 individual CNV calls (1167 copy number gain calls, 30 copy number loss calls), were not mapped to the new build as the sequence they represented from build35 was either partially deleted or split across more than region in build 36.

### Statistical Analysis

The Pearson's chi-squared test (1 degree of freedom) was used for comparison of the number of ALS-specific and the number of control-specific CNV loci. A two-tailed Fisher's exact test was used to test each autosomal CNV locus for differences between cases and controls – losses and gains at a single locus were considered together. A two-tailed Fisher's exact test was also used for gene-based association testing with losses and gains considered both separately and together. Mann-Whitney U-tests were used for comparison of CNV size and mean number per individual between cases and controls. No multiple testing corrections were applied. A significance threshold of P<0.05 was used.

### Pathway Analysis

#### Gene Set analysis

Gene Set analysis (GSA) was undertaken using the GenGen package [49] which implements the Gene-Set Enrichment Analysis introduced by Subramanian, Tamayo et al [50]. Significance was estimated empirically by permutation of the phenotype labels (1000 permutations). All genes from Ensembl v54 (www.ensembl.org) that were overlapped by CNV regions defined using a 70% reciprocal overlap threshold (plus 20 kb upstream and downstream) were included in the analysis and the association p-value from the overlapping CNV region was assigned to the gene. We extended the coordinates by 20 kb as per Veyrieras *et al*. (2008) who showed that approximately 95% of common SNPs likely to affect gene expression reside in the proximal 20 kb to the transcription start and end sites [51]. A total of 115 KEGG and Gene Ontology level 4 gene sets containing between 20 and 200 genes were tested. Since many gene sets are redundant, the effective number of pathways was calculated using an approach previously proposed to calculate the effective number of tests for genotype data [52]. A total of 87 effective pathways were tested with a threshold of 5.74×10^-4^ defined by Bonferroni correction for significance.

This analysis was also carried out using p-values from the gene-based association testing. A total of 105 pathways that included 97 effective pathways was tested and a threshold for significance of 5.15×10^−4^ was defined.

#### GRAIL

A GRAIL analysis [53] was carried out on ALS-specific (n = 119) and control-specific (n = 136) CNV calls independently. The seed regions (regions to be tested) were set the same as the query region (in this case the CNV call coordinates) due to the absence of any previously identified CNV regions strongly associated with ALS. In brief, for each gene overlapped by a query region all other human genes are given a relatedness ranking using a similarity measure calculated by text mining of PubMed abstracts. A count of all genes within all seed regions that have a relatedness ranking less than a given threshold (which is dependent on the number of genes within the seed region) for the candidate gene is then obtained and a p value for the count calculated. The best scoring gene within each query region is then identified using the p value corrected for multiple testing according to the number of candidate genes within the query region and any genes that reach significance (p<0.05) are reported. The reported genes are the genes with the most number of relationships to other genes in independent associated regions.

## Supporting Information

Appendix S1 Supplementary Methods: Simulations to define filtering thresholds and alternative CNV call filtering strategy for comparison with previous study.
(0.03 MB DOC)

Table S1 CNV regions showing significant association with ALS but coordinates not transferable to build 36.
(0.03 MB DOC)

Table S2 Summary of all association results (p<0.05 using Fishers exact test).
(0.07 MB DOC)

Table S3 Pathways nominally associated with ALS (P<0.05) using GSA analysis. Both region-based and gene-based association statistics were used.
(0.04 MB DOC)

Table S4 Result from GRAIL analysis of ALS-specific CNV calls and control-specific CNV calls (corrected p<0.05).
(0.03 MB DOC)

Figure S1 Sensitivity and false positive rate for varying thresholds of the log Bayes factor (simulation). Receiver Operating Curve (ROC) curve showing sensitivity of CNV calls (1 - proportion of false negative calls) and genome-wide number of false positive calls for varying thresholds of the log Bayes factor. Log Bayes factor thresholds up to >6 are indicated on the plot.
(0.33 MB EPS)
