# Supplementary material for: The Role of Copy Number Variation in Susceptibility to Amyotrophic Lateral Sclerosis: Genome-Wide Association Study and Comparison with Published Loci
Source: PLoS One. 2009 Dec 4;4(12):e8175. doi: 10.1371/journal.pone.0008175 (PMC2780722; doi:10.1371/journal.pone.0008175)
Supplement: Appendix S1 — Supplementary Methods: Simulations to define filtering thresholds and alternative CNV call filtering strategy for comparison with previous study. (0.03 MB DOC) [file pone.0008175.s001.doc]

**Supplementary methods**

*Simulations*

Simulations were carried out by randomising the log R ratios and B allele frequencies amongst all SNPs across chromosome 1 from a male control sample. This sample was chosen as it passed all QC criteria recommended by the program authors and did not contain an unusually high number of putative CNVs (as detectable by QuantiSNP) across the genome before randomisation, especially on chromosome 1. This was repeated 1000 times. QuantiSNP was then run on each of these simulated samples to give an estimate of the false positive rate.

We assessed the sensitivity of the CNV calls by utilising SNP data from male chromosome X segments; as these are hemizygous genotypes they were used to represent deletions. We simulated deletions of varying lengths (ranging from 1 to 40 SNPs) from randomised chromosome X segments from the same male control sample. One deletion CNV was inserted into each randomised chromosome 1 such that each CNV length was simulated 1000 times. To obtain rates per genome, it was assumed that chromosome 1 constitutes 8% of the genome and the chromosomal rate was scaled accordingly.

Figure S1 shows that relaxing the LBF filter from >6 to >4 for simulated deletions of any length improved sensitivity, but resulted in an increase in the false positive rate. There was better sensitivity to detect longer CNVs (of greater SNP lengths).

*Alternative CNV call filtering strategy for comparison with previous study*

For the purposes of a more direct comparison with the results of a previous study[1], the CNV calls from QuantiSNP were also filtered in a similar way to those from the previous study. Samples were excluded if they exhibited a LRR standard deviation of >0.3 or a BAF standard deviation of >0.15. Calls with a LBF of <10 were discarded. Again, CNVs that spanned the centromeres and CNVs from samples with high numbers of calls across their genome were excluded.

**References**

1. Cronin S, Blauw HM, Veldink JH, van Es MA, Ophoff RA, et al. (2008) Analysis of genome-wide copy number variation in Irish and Dutch ALS populations. Hum Mol Genet 7: 7.
