# Supplementary material for: The Role of Copy Number Variation in Susceptibility to Amyotrophic Lateral Sclerosis: Genome-Wide Association Study and Comparison with Published Loci
Source: PLoS One. 2009 Dec 4;4(12):e8175. doi: 10.1371/journal.pone.0008175 (PMC2780722; doi:10.1371/journal.pone.0008175)
Supplement: Table S1 — CNV regions showing significant association with ALS but coordinates not transferable to build 36. (0.03 MB DOC) [file pone.0008175.s002.doc]

| chr | start | end | ALS | controls | P value | Distance from centromere (kb) | Genes | CNP1 | freq |
| --- | --- | --- | --- | --- | --- | --- | --- | --- | --- |
| 11 | 49824461 | 51297186 | 254 | 205 | 3.75x10^-4^ | 153.6 | - | 1 | 0.384 |
| 7 | 60882694 | 61848856 | 4 | 19 | 0.0016 | 17.7 | - | 1 | 0.019 |
| 16 | 33744011 | 35106851 | 14 | 2 | 0.0036 | 36.5 | UBE2MP1 | 2 | 0.013 |
| 6 | 58183439 | 58276111 | 1 | 13 | 0.0045 | 662 | - | 0 | 0.012 |
| 6 | 57988851 | 58878583 | 1 | 11 | 0.0065 | 59.5 | GUSBL2 | 1 | 0.010 |
| 7 | 60882694 | 62282682 | 128 | 97 | 0.0090 | 17.7 | LOC643955 | 2 | 0.188 |

All coordinates and genes are from build 35. All regions contained copy number gains only.
