# Supplementary material for: The Role of Copy Number Variation in Susceptibility to Amyotrophic Lateral Sclerosis: Genome-Wide Association Study and Comparison with Published Loci
Source: PLoS One. 2009 Dec 4;4(12):e8175. doi: 10.1371/journal.pone.0008175 (PMC2780722; doi:10.1371/journal.pone.0008175)
Supplement: Table S2 — Summary of all association results (p<0.05 using Fishers exact test). (0.07 MB DOC) [file pone.0008175.s003.doc]

| **Chr** | **Start (bp)** | **End (bp)** | **ALS** | **controls** | **P value** | **Distance from centromere (kb)** | **Overlapped genes** | **CNP1** | **Frequency across all samples** |
| --- | --- | --- | --- | --- | --- | --- | --- | --- | --- |
| ***Gains*** | | | | | | | | | |
| 5 | 45850032 | 46384240 | 264 | 174 | 1.07x10^-9^­ | 324.3 | - | 2 | 0.367 |
| 8 | 47062007 | 47406312 | 30 | 8 | 8.21x10^-5^ | 276.1 | - | 0 | 0.032 |
| 12 | 36528296 | 36801139 | 157 | 109 | 2.65x10^-4^ | 521.8 | - | 1 | 0.223 |
| 19 | 32615675 | 32935836 | 165 | 122 | 5.87x10^4^ | 2852.1 | RDH13 | 0 | 0.240 |
| 7 | 61663407 | 62155064 | 172 | 132 | 0.0018 | 851.0 | - | 0 | 0.255 |
| 3 | 33270957 | 33296620 | 3 | 18 | 0.0027 | 57303.8 | *FBXL2* | 0 | 0.018 |
| 8 | 47062007 | 47711911 | 3 | 18 | 0.0032 | 428.9 | - | 1 | 0.018 |
| 8 | 43689385 | 43910848 | 74 | 52 | 0.0046 | 157.9 | - | 0 | 0.106 |
| 16 | 969913 | 1834962 | 8 | 15 | 0.0056 | 33740.9# | SOX8, SSTR5, C1QTNF8, **CACNA1H**, TPSG1, TPSB2, TPSAB1, TPSD1, **UBE2I**, **BAIAP3**, C16orf42, GNPTG, UNKL, C16orf91, CLCN7, C16orf38, TELO2, IFT140, TMEM204, CRAMP1L, HN1L, **MAPK8IP3**, NME3, MRPS34, EME2, **SPSB3**, NUBP2, **IGFALS**, HAGH, FAHD1, C16orf73 | 1 | 0.019 |
| 5 | 28842013 | 28912873 | 7 | 0 | 0.0058 | 17563.9 | - | 0 | 0.006 |
| 4 | 761587 | 1014752 | 16 | 4 | 0.0068 | 48466.7# | **CPLX1**, GAK, TMEM175, DGKQ, *IDUA*, *SLC26A1, FGFRL1* | 0 | 0.017 |
| 5 | 510955 | 738748 | 6 | 0 | 0.012* | 45816.6# | EXOC3, FLJ00157, CR610608, SLC9A3, CEP72, TPPP | 0 | 0.005 |
| 20 | 60320976 | 60493125 | 0 | 8 | 0.016 | 32373.8 | *LAMA5, RPS21, CABLES2, C20orf151, GATA5* | 0 | 0.007 |
| 19 | 32615675 | 32851754 | 1 | 10 | 0.022 | 2810.1 | - | 0 | 0.009 |
| 6 | 58675121 | 58878583 | 1 | 10 | 0.024 | 161.3 | - | 0 | 0.009 |
| 19 | 60270514 | 60293927 | 5 | 1 | 0.025 | 30358.6 | EPS8L1 | 0 | 0.005 |
| 14 | 103232016 | 103721150 | 5 | 0 | 0.025 | 85406.6# | KLC1, XRCC3, ZFYVE21, ***PPP1R13B****, C14orf2*, TDRD9, ASPG, KIF26A | 0 | 0.004 |
| 20 | 60214968 | 60493125 | 10 | 23 | 0.035 | 32320.8# | HRH3, OSBPL2, ADRM1, *LAMA5*, *RPS21, CABLES2, C20orf151, GATA5* | 0 | 0.028 |
| 16 | 1744358 | 1781034 | 11 | 3 | 0.038 | 33380.6# | **MAPK8IP3**, NME3, MRPS34, EME2, **SPSB3**, NUBP2, **IGFALS** | 0 | 0.012 |
| 14 | 104197399 | 104356204 | 10 | 2 | 0.043* | 86206.8 | INF2, ADSSL1, SIVA1, AKT1, ZBTB42 | 0 | 0.010 |
| 8 | 142512205 | 142529990 | 38 | 22 | 0.046* | 95563.1# | FLJ43860 | 0 | 0.050 |
| 3 | 101837214 | 101916282 | 5 | 10 | 0.047 | 8389.2 | GPR128, TFG | 0 | 0.013 |
| 21 | 43646295 | 43663581 | 10 | 4 | 0.047 | 30394.9# | SIK1 | 1 | 0.012 |
| 14 | 19375271 | 19536664 | 20 | 41 | 0.049 | 1386.0 | OR4K1, OR4K2, OR4K5, OR4K15 | 1 | 0.051 |
| 11 | 382079 | 434628 | 11 | 10 | 0.049 | 51042.4# | PKP3, SIGIRR, ANO9 | 0 | 0.018 |
| ***Losses*** | | | | | | | | | |
| 22 | 21011312 | 21394287 | 0 | 11 | 0.002 | 6872.8 | *ZNF280B, ZNF280A, PRAME, BCR,* GGTLC2, | 0 | 0.009 |
| 10 | 82869699 | 82882268 | 7 | 22 | 0.013 | 41251.0 | - | 1 | 0.024 |
| 11 | 539119 | 652407 | 5 | 0 | 0.025 | 50855.0# | *LRRC56, C11orf35, RASSF7, PHRF1*, *IRF7, MUPCDH*, *SCT,* *DRD4*, *DEAF1* | 0 | 0.004 |
| 17 | 43969101 | 44059535 | 7 | 1 | 0.032 | 21727.2 | HOXB2, HOXB3, HOXB4, HOXB5, HOXB6, HOXB7, HOXB8, HOXB9 | 0 | 0.007 |
| ***Losses / Gains*** | | | | | | | | | |
| 8 | 144686338 | 144765210 | 6 | 0 | 0.012 | 97767.7# | ZC3H3, *GSDMD*, *C8orf73*, *NAPRT1,* ***EEF1D***, TIGD5, PYCRL | 0 | 0.005 |
| 8 | 47224322 | 47711911 | 8 | 20 | 0.039 | 510.1 | - | 1 | 0.023 |

A reciprocal overlap threshold of >70% was used. 1 Number of CNPs from the McCarroll CNP map [37] that also overlap this region. * This region was not significant (p<0.05) if other reciprocal overlap thresholds were used. # Regions that were within the telomeric chromosome band. Seven additional regions identified to show significant association with ALS were unable to be mapped to build 36 of the human genome. These regions are given in Error: Reference source not found. Genes which were also identified with p<0.01 in the gene-based analysis are given in italics. Genes which may be reasonable ALS candidates are in bold.
