# Supplementary material for: The Role of Copy Number Variation in Susceptibility to Amyotrophic Lateral Sclerosis: Genome-Wide Association Study and Comparison with Published Loci
Source: PLoS One. 2009 Dec 4;4(12):e8175. doi: 10.1371/journal.pone.0008175 (PMC2780722; doi:10.1371/journal.pone.0008175)
Supplement: Table S3 — Pathways nominally associated with ALS (P<0.05) using GSA analysis. Both region-based and gene-based association statistics were used. (0.04 MB DOC) [file pone.0008175.s004.doc]

| GO/KEGG id | Pathway description | Number of genes | nominal p |
| --- | --- | --- | --- |
| **Region-based** | | | |
| GO0042981 | Regulation of apoptosis | 78 | 2.00E-03 |
| GO0005996 | Monosaccharide metabolic process | 22 | 4.00E-03 |
| GO0007517 | Muscle organ development | 25 | 4.00E-03 |
| GO0012502 | Induction of programmed cell death | 35 | 5.00E-03 |
| GO0043065 | Positive regulation of apoptosis | 45 | 9.00E-03 |
| GO0034984 | Cellular response to DNA damage stimulus | 39 | 1.00E-02 |
| GO0004553 | Hydrolase activity, hydrolyzing O-glycosyl compounds | 24 | 1.70E-02 |
| GO0006520 | Cellular amino acid metabolic process | 29 | 1.80E-02 |
| hsa04010 | MAPK signaling pathway | 30 | 2.20E-02 |
| GO0007417 | Central nervous system development | 28 | 2.70E-02 |
| GO0043066 | Negative regulation of apoptosis | 31 | 2.80E-02 |
| GO0051246 | Regulation of protein metabolic process | 33 | 3.20E-02 |
| GO0032268 | Regulation of cellular protein metabolic process | 26 | 3.40E-02 |
| hsa04530 | Tight junction | 26 | 3.80E-02 |
| **Gene-based** | | | |
| GO0007156 | Homophilic cell adhesion | 22 | 1.000e-03 |
| GO0007517 | Muscle organ development | 23 | 1.800e-02 |
| GO0008168 | Methyltransferase activity | 20 | 3.700e-02 |
