# Supplementary material for: The Role of Copy Number Variation in Susceptibility to Amyotrophic Lateral Sclerosis: Genome-Wide Association Study and Comparison with Published Loci
Source: PLoS One. 2009 Dec 4;4(12):e8175. doi: 10.1371/journal.pone.0008175 (PMC2780722; doi:10.1371/journal.pone.0008175)
Supplement: Table S4 — Result from GRAIL analysis of ALS-specific CNV calls and control-specific CNV calls (corrected p<0.05). (0.03 MB DOC) [file pone.0008175.s005.doc]

| Candidate Gene | p | Connected genes (relatedness ranking*) |
| --- | --- | --- |
| ALS-specific CNV call analysis | | |
| None | - | - |
| Control-specific CNV call analysis | | |
| MEIG1 | 0.005086023 | STRA8(6), YBX2(13), ANKRD36(26), DPPA3(28), FAM75A5(148), TUBB2C(216), PCSK4(241), GDF3(352), BRD3(354), TUBA3D(404), PHPT1(432), HN1L(508), PNPLA7(567), AKAP12(595) |
| FAM75A5 | 0.024288605 | ANKRD36(2), MEIG1(12), STRA8(74), PCSK4(182), BRD3(279), KIAA1688(290), HN1L(299), CACNA1H(308), POLR2A(493), TUBB2C(596) |
| IGLL1 | 0.025608492 | VPREB1(2), ZNF280A(10), ZNF280B(14), CRYL1(46), HLA-B(87), HN1L(93), BRD3(170), GGTLC2(239), PHPT1(288), POLR2A(351), TUBB2C(588) |
| STRA8 | 0.035710229 | MEIG1(6), YBX2(26), DPPA3(27), C17orf81(99), BRD3(142), GDF3(192), TUBB2C(224), NPDC1(289), ANKRD36(314), TUBA3D(368), PHPT1(439), HN1L(501), RPS21(605), AKAP12(611) |
| ANKRD36 | 0.012931892 | FAM75A5(7), MEIG1(9), LRP2BP(74), STRA8(116), PCSK4(174), YBX2(218), TEKT4(268), PHF23(376), HN1L(507) |

***Always >=2; the candidate gene itself is rank (1).**
